# A Prognostic and Carboplatin Response Predictive Model in Ovarian Cancer: A Mono-Institutional Retrospective Study Based on Clinics and Pharmacogenomics

**DOI:** 10.3390/biomedicines10051210

**Published:** 2022-05-23

**Authors:** Nicoletta Staropoli, Mariamena Arbitrio, Angela Salvino, Francesca Scionti, Domenico Ciliberto, Rossana Ingargiola, Caterina Labanca, Giuseppe Agapito, Eleonora Iuliano, Vito Barbieri, Maria Cucè, Valeria Zuccalà, Mario Cannataro, Pierfrancesco Tassone, Pierosandro Tagliaferri

**Affiliations:** 1 Medical Oncology Unit, AOU Mater Domini, 88100 Catanzaro, Italy; angsalvino@yahoo.it (A.S.); cilibertodomenico@hotmail.com (D.C.); mariacuce1@gmail.com (M.C.); tassone@unicz.it (P.T.); 2 Institute for Biomedical Research and Innovation (IRIB), National Research Council of Italy (CNR), 88100 Catanzaro, Italy; 3 Institute for Biomedical Research and Innovation (IRIB), National Research Council of Italy (CNR), 98125 Messina, Italy; francesca.scionti@irib.cnr.it; 4 Department of Experimental and Clinical Medicine, Magna Græcia University, 88100 Catanzaro, Italy; rossana.ingargiola@cnao.it (R.I.); caterinalabanca87@gmail.com (C.L.); eleonorafiuliano@hotmail.com (E.I.); 5 Department of Law, Economics and Sociology, Magna Graecia University of Catanzaro, 88100 Catanzaro, Italy; agapito@unicz.it; 6 Data Analytics Research Center, Magna Graecia University of Catanzaro, 88100 Catanzaro, Italy; cannataro@unicz.it; 7 Medical Oncology Unit, “Pugliese-Ciaccio” Hospital, 88100 Catanzaro, Italy; vitobarbieri@yahoo.it; 8 Pathology Unit, “Pugliese-Ciaccio” Hospital, 88100 Catanzaro, Italy; valezy@libero.it; 9 Department of Medical and Surgical Sciences, Magna Graecia University of Catanzaro, 88100 Catanzaro, Italy

**Keywords:** advanced ovarian cancer, targeted therapy, carboplatin, DMET analysis, prognostic factors

## Abstract

Carboplatin is the cornerstone of ovarian cancer (OC) treatment, while platinum-response, dependent on interindividual variability, is the major prognostic factor for long-term outcomes. This retrospective study was focused on explorative search of genetic polymorphisms in the Absorption, Distribution, Metabolism, Excretion (ADME) genes for the identification of biomarkers prognostic/predictive of platinum-response in OC patients. Ninety-two advanced OC patients treated with carboplatin-based therapy were enrolled at our institution. Of these, we showed that 72% of patients were platinum-sensitive, with a significant benefit in terms of OS (*p* = 0.001). We identified an inflammatory-score with a longer OS in patients with lower scores as compared to patients with the maximum score (*p* = 0.001). Thirty-two patients were genotyped for 1931 single nucleotide polymorphisms (SNPs) and five copy number variations (CNVs) by the DMET Plus array platform. Among prognostic polymorphisms, we found a potential role of UGT2A1 both as a predictor of platinum-response (*p* = 0.01) and as prognostic of survival (*p* = 0.05). Finally, we identified 24 SNPs related to OS. UGT2A1 correlates to an “inflammatory-score” and retains a potential prognostic role in advanced OC. These data provide a proof of concept that warrants further validation in follow-up studies for the definition of novel biomarkers in this aggressive disease.

## 1. Introduction

Epithelial ovarian cancer (OC) is the fifth cause of female death, with a lifetime risk of developing it in 1/75 women [1,2]. The doublet of carboplatin/paclitaxel still represents the gold standard of treatment. Platinum salts are the gold standard of OC therapy in early high-risk and advanced stages of OC over the past 40 years worldwide. Several studies demonstrated the superiority of regimens including carboplatin over cisplatin for tolerability and efficacy. Advanced-recurrent disease usually receives a second-line chemotherapy, selected according to previous platinum response (platinum-free interval, PFI) [3,4,5,6,7,8,9]. Platinum response is a multifactorial status associated with genetic and non-genetic risk factors, many of which are unknown. So far, despite several investigations, no molecular and genomic alterations have been identified as predictive factors of platinum refractories. In this scenario, the early loss of BRCA and TP53 functions was strongly associated with platinum-response [10]. This last observation was described experimentally in in vitro and in vivo models demonstrating the failure of the DNA repair homologous recombination mechanisms (due to loss of BRCA1-2 function) to restore double-strand breaks induced by platinum compounds. This condition, like hereditary/somatic mutations in BRCA1-2, correlated to a good response to platinum-chemotherapy [11,12,13,14]. When platinum-sensitivity is considered in addition to family history and visceral involvement, with or without germinal/somatic BRCA mutations, HGSOC is commonly defined as the “BRCA-ness phenotype”. Recently, it has been demonstrated that patients with wild-type BRCA (BRCAwt) have a copy number deletion of RAD50 or HR deregulation (HRD). This deletion seems to represent a predictive marker of “BRCA-ness phenotype” [15,16]. These patients are often characterized by a better survival outcome and a BRCA-independent mechanism of drug sensitivity [17]. Moreover, in the onset and development of OC, several studies investigated the role of inflammation, immune landscape, and angiogenesis in malignant cells and their microenvironment, which have been linked to poor prognosis, advanced disease stage, residual disease status, and ascites [18,19]. At diagnosis, the majority of OC patients present ascites and/or peritoneal involvement [20]. Tumor infiltration by different inflammatory cells has been correlated with prognosis and tumor progression in OC [21]. Furthermore, several findings demonstrated that tumor-infiltrating lymphocytes, the high mutational burden in the tumor genome, and alterations of mismatch repair genes may represent important predictive factors of efficacy of immune checkpoints inhibitors, but the real impact of these factors in OC remains to be elucidated. Also, vaccines, cytokines, and adoptive T-cell therapy have been hypothesized as immune-based approaches in the development of OC treatment [22]. A meta-analysis suggested that neutrophil to lymphocyte ratio (NLR), the platelet to lymphocyte ratio (PLR), and plasmatic fibrinogen level could represent important prognostic biomarkers in OC as the expression of the inflammation process, particularly in an advanced stage of disease [23]. Moreover, inter-individual variability in platinum response could also be due to germline variants in genes involved in drug absorption, distribution, metabolism, and excretion (ADME) [24,25,26].

In this study, we performed integrated analyses of clinical, laboratory, and molecular data on genomic variants for the identification of potential prognostic biomarkers associated with predictive platinum response in OC patients. Moreover, in a randomly selected group of 32 patients, the role of 1931 ADME markers and 5 Copy Number Variations (CNV) was investigated for the identification of potentially predictive biomarkers correlated to interindividual variability in platinum response [27].

## 2. Materials and Methods

### 2.1. Patients

To set up a database on OC patients, we performed a retrospective analysis on 92 patients followed at our institution in the time-frame 2006–2018. Clinical data have been selected from medical records preserved in institutional archives. Surgical specimens were retrieved from the archive files of the Department of Health Sciences, Surgical Pathology Section of the University Magna Græcia of Catanzaro, Italy. In this contest, for the time-frame 2014–2016, regarding consecutive 32 patients, blood samples were collected for DMET plus genotyping after Ethical Committee approval, and informed consent was obtained from all patients in accordance with the Recommendations of the Declaration of Helsinki for biomedical research involving human subjects [28]. The study was compliant with institutional bioethical standards. This study was designed according to recommendations for REMARK criteria.

Eligibility criteria. These included the histologically-confirmed diagnosis of ovarian adenocarcinoma, age > 18 years, ECOG performance Status 0–1, adequate renal and liver function, and no major comorbidities.

Exclusion criteria. These included other malignancies, no basal data retrievable for first-line treatment (e.g., diagnosis previous to 2006 or chemotherapy performed in another centre).

All enrolled patients had a high-grade serous or endometrioid tumour (only 10%) and an advanced stage of disease (III and IV stage). All patients were treated and followed up exclusively at our institution and received platinum-based therapy. In our series, we evaluated different OC parameters identified as prognostic factors: age (y), performance status Eastern Cooperative Oncology Group (PS ECOG), the start of diagnosis, stage at diagnosis, the presence of metastases, CA125 levels (baseline, during chemotherapy, at eventual disease relapse), first-line chemotherapy, response to treatment and toxicity reports, eventual other surgery, and line of chemotherapy subsequently performed.

Response Evaluation Criteria in Solid Tumors (RECIST 1.1) was used to define the response to chemotherapy. As well as institutional clinical practice, we monitored patient outcomes every three months. For increasing CA125 or clinical symptoms, patients underwent imaging procedures such as a total body count tomography scan (TC scan) to assess the status of the disease. Patients were grouped as PR (platinum-resistant) and PS (platinum-sensitive), taking into account the classification system based on the PFI.

### 2.2. Molecular Analysis

Within the specific time frame of December 2016–January 2018, consecutive peripheral blood samples from 32 patients with OC was collected at our institution and genotyped by DMET Plus assay (Thermo Fisher Scientific Inc., Waltham, MA, USA), including 1931 SNPs and five CNVs in ADME genes [29,30] as previously described in common and uncommon diseases [31,32,33,34].

### 2.3. Bioinformatics and Statistical Analysis

DMET Console software was used to perform genotype calls from intensity array data. Genotypes with a call rate < 95% were excluded. Association analysis between platinum response and genotypes was performed by Fisher’s test [35] and the Bonferroni corrector to adjust the computed p-values; both methods are available in DMET-Analyzer software [36]. In [35], the authors present a method to compare responders (relapse obtained after 12 months by last carboplatin cycle) vs. non responders (if relapse occurs within six months) according to PFI. Starting with this first analysis, it was possible to identify 23 particular genotypes in LD with specific significant SNPs related to platinum sensitivity. Thus, clinical and laboratory parameters were compared to significant genotypes in order to evaluate the prognostic role. Preliminary power estimation to detect the association between SNPs and platinum response was performed using the Power of Genetic Analysis (PGA) package. In order to avoid selection bias, data were collected by two independent investigators (N.S. and R.I.) and the missing information has been discussed and solved with the aid of arbiters (M.A. and D.C.). Primary endpoints were OS and PFS (to define a possible prognostic role of different parameters). The secondary endpoint was response rate (RR) with respect to platinum-sensitivity (regarding the advanced stage of disease); the time elapsed between the start of treatment and the date of death describes the OS. PFS is reported as the time from the start of treatment to progression or death. A Wilcoxon test and a chi-square test were used for the analyses regarding the differences between the patients’ baseline characteristics.

For each comparison between two patient groups for a major identified factor we used a Student’s t-test. The major variables considered in this work are dichotomized on median value. The effect of several variables on outcome was evaluated with Kaplan-Meier curves and a Log Rank test. *p*-value < 0.05 defines a statistically significant result. The relative HR with 95% confidence intervals (95% CIs) were calculated using the IBM Statistical Package for Social Sciences (SPSS) version 22 (IBM Corp, Armonk, NY, USA), Graphpad PRISM version 6.0 (GraphPad Software, San Diego, CA, USA), and the R Statistical Software (v3.4.2; R Core Team 2017).

## 3. Results

In this work, we performed a retrospective mono-institutional analysis. Ninety-two patients with OC were treated at the Oncology Unit, University Mater Domini Teaching Hospital of Catanzaro from January 2006 to January 2018 (Table 1). Age, performance status, and stage at diagnosis were comparable to inclusion criteria of major trials; the median age was 59 years, median ECOG was PS 0.88% of patients presented with advanced-stage (FIGO III–IV). At diagnosis, ascites and peritoneal involvement were observed in 53% and 64% of patients, respectively. All were high-grade serous OC (HGSOC) or high-grade endometrioid tumours. The median value of basal CA 125 was 377 U/mL in the whole study population, and the patients were divided into two groups according to this cut-off.

Fifty-one percent of the patients received a second-line treatment, and about 25% underwent a third-line treatment; 20% of the patients underwent second-look surgery. The median OS in patients in the early stage of the disease was 80 months, with a better outcome as compared to data in the literature. Fifty-four percent of patients presented a BRCAness phenotype according to the clinical parameters previously described. Thirty-four (37%) patients underwent NGS analysis for the definition of BRCA status. Of these, in 10 of the 34 (30%) patients, a pathogenic mutation was found (in detail we observed two BRCA1 deletions in Exon 11c.1360_1361del.AG and one in Exon16c.4964_4982del.19; one BRCA1 Exon11c.2722G > T nonsense; one BRCA1 Exon5_c.181T > G missense and BRCA2 Exon11_c.5593A > T missense; four BRCA1 mutations not detailed); in five, observed mutations presented a clinical relevance (class 5 pathogenic mutation); in one, mutation was a class 3 VUS. All patients received carboplatin-paclitaxel as first-line treatment and 23 patients also received bevacizumab. According to the PFI definition, all patients were split into platinum-sensitive (46 patients, 65%) and platinum-resistant (25 patients, 35%). Considering our database, it was possible to retrieve data about pre-treatment laboratory findings (i.e., neutrophil, lymphocyte, and platelet counts; fibrinogen, d-dimer, alkaline phosphatase, lactic dehydrogenase, albumin). Patients were divided into two groups based on median value as cut-off) >6700 vs. <6700 cells/mm3 neutrophils count (upper limit of normal), >1275 vs. <1275 cells/mm^3^ lymphocytes count (lower limit of normal), >400 × 103 vs. <400 × 103 cells/mm^3^ platelets count (upper limit of normal), <6 vs. >6 NLR (measured as the ratio between neutrophil and lymphocyte counts at baseline) (mean of the group); we also evaluated <3 vs. >3 NLR as value previously reported in scientific literature; <244 vs. >244 PLR (measured as the ratio between platelet and lymphocyte counts at baseline) (mean of the group) (Table 2).

**Figure 1:**
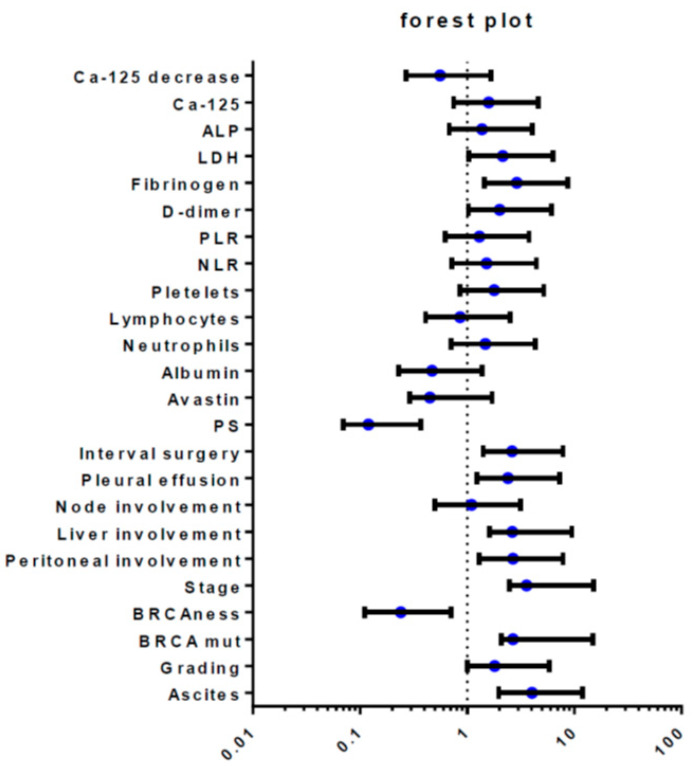
Forest Plot, major prognostic factors; Figure shows the effect of univariate analysis for each prognostic factor by log rank test for each variable, reported with appropriate confidence interval.

**Figure 2:**
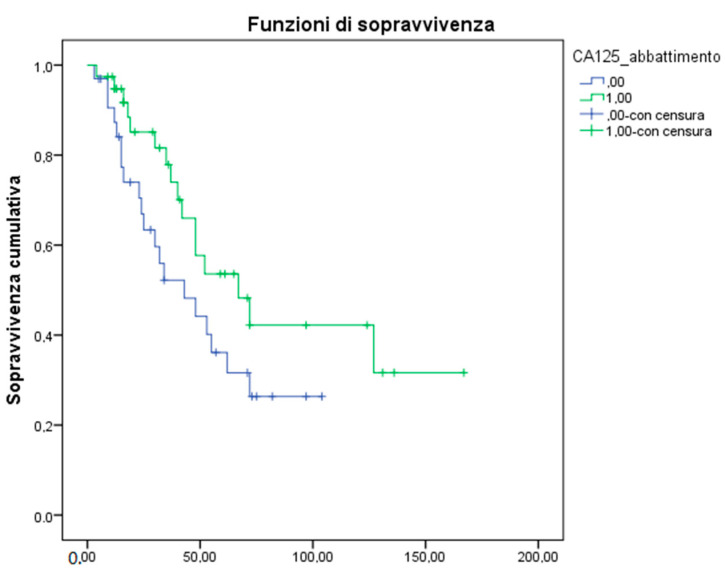
CA 125 decrease in terms of Overall Survival (OS). The median value of basal CA 125 was 377 U/mL in the whole study population and it was calculated on the basis of this cut-off. Blue line: Missing decrease of CA 125 value with respect to basal value. Green line: reset of CA 125 value with respect to basal value.

**Figure 3:**
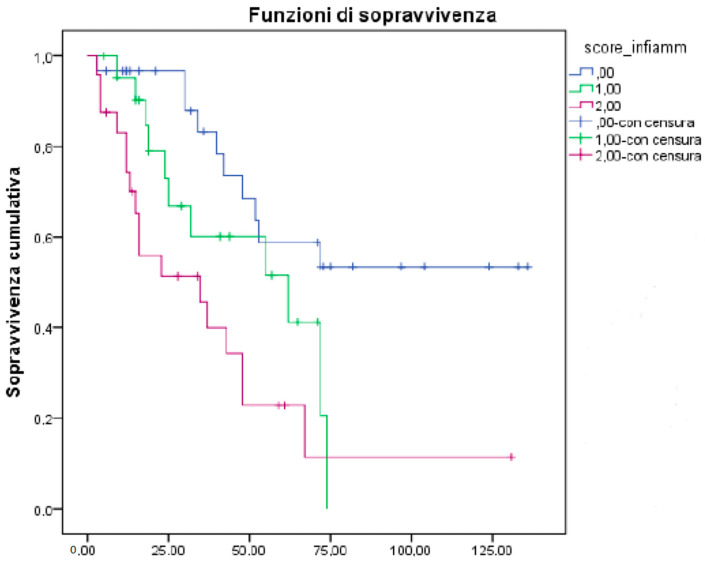
Platinum status in correlation to OS. PFI > 12 months (blue line); PFI 6–12 months (green line); PFI <12 months (purple line).

The median Overall Survival (OS) was 55 months for all EOC patients, 48 months for those in the advanced-stage of the disease. Median Progression-Free Survival (PFS) was 18 months in the advanced stage. All potential prognostic factors were transformed into categorical variables, dichotomous on a median value. Thus, we investigated the impact of each prognostic factor on survival in a univariate analysis by Log Rank test, Kaplan- Meier descriptive statistics, and a Cox Proportional Hazard ratio model. Starting with these results, we confirmed the role of stage at diagnosis (*p* = 0.0001) and platinum-sensitivity (*p* = 0.00001), which are probably the major prognostic factors (Table 3). Moreover, we observed that node involvement did not produce any statistically significant difference in terms of OS (Hazard ratio, HR 0.74), but the presence of peritoneal carcinomatosis and/or abdominal involvement correlated with a worse survival outcome. In detail, patients who presented ascites (HR 4.04), pleural effusion (HR 2.4) liver (HR 2.64), or peritoneal involvement (HR 2.68) at diagnosis have a significantly lower OS as compared to the absence of these conditions. (Figure 1). Taking into account that an important correlate of effusion is the presence of abnormal CA 125 level at diagnosis, we found that patients with the lower CA 125 value at diagnosis had a better outcome in terms of PFS (HR 0.40; CI 0.21 to 0.79, *p* = 0.008) that translated in a potential trend in OS (HR 0.14; *p* = 0.14). We also confirmed a role of CA 125 decrease after first-line treatment with an OS of 67 months (HR 0.56) (Figure 2). According to platinum-sensitivity, the median OS was 30 months for refractory patients, 80 months for patients partially platinum-sensitive, while median survival was still not reached for platinum-sensitive patients (HR 0.12; median OS of total PS 94 months). This last subset showed the most significant advantage in terms of OS, *p* = 0.001. (Figure 3). We explored the BRCAness phenotype in our population, demonstrating that these patients showed a significant advantage both in terms of OS (HR 0.25) and PFS (*p* = 0.00013). We searched the germline mutation in a small percentage of cases because this study design was prior to the current Italian recommendations [37], and the short follow-up could not permit the identification of the relevance of genetic background.

However, with regard to germline BRCA analysis, we showed that the small sample size, as highlighted by larger IC (0.59–12.22), did not permit the achievement of a reliable result, and thus our findings do not reflect the real-world data on this parameter.

### 3.1. DMET^TM^ plus Allowed the Identification of Potential Pharmacogenomic Predictive/Prognostic Biomarkers Correlated to Platinum Response

In Table 4 we report the results obtained from association analysis in which a Fisher’s test demonstrated an association between ADME genotypes indicated by SNPs in linkage disequilibrium with specific polymorphic variants and platinum-sensitivity. Particularly, we selected the SNPs according to Bonferroni correction, reporting a *p*-value < 0.00313. Matching the clinical and pathological characteristics, we identified 20 genotypes correlated to survival outcome and platinum-response. Moreover, among the polymorphic variants correlated to platinum-sensitivity, we highlighted the *UGT2A1*_57885 > (rs2288741) for its association both as platinum-response predictor (*p* = 0.0001) and as a prognostic biomarker (*p* = 0.05).

### 3.2. Inflammatory Status

To identify basal hematological conditions related to inflammatory status with platinum-sensitivity, we collected pre-treatment laboratory findings. Particularly, we observed a worse OS in patients with a lower albumin level (HR 0.47), high levels of fibrinogen (HR 2.9), D-dimer (HR 2.01), LDH (HR 2.15), and ALP (HR 1.38). A D-dimer value in the normal range was associated with a longer PFS (28 vs. 13 months; HR 0.35 CI: 0.06 to 1.77, *p* = 0.07). We considered the most significant laboratory parameters identified through multivariate analysis (albumin, D-dimer, fibrinogen, LDH, ALP, platelet count, neutrophil count, lymphocyte count) in correlation to platinum-sensitivity and survival outcome (Table 5).

Moreover, we observed a significant difference in neutrophil count (HR 1.48) and platelet count (HR 1.79), but no OS difference was found concerning lymphocyte count. On this basis, we evaluated both NLR and PLR on median value cut off, and we observed a trend vs. better OS in patients with lower NLR (*p* = 0.19), while PLR did not represent a prognostic factor. For platinum status, PR (platinum-resistant) patients had neutrophil and platelet counts significantly higher compared to PS (platinum-sensitive) patients. Furthermore, regarding platinum-sensitivity, we showed a strong correlation (by chi-square analysis) with ascites (*p* = 0.02), peritoneal involvement (*p* = 0.030), albumin (*p* = 0.019), platelet count (*p* = 0.034), fibrinogen (*p* = 0.01), and a potential trend with NLR (*p* = 0.06) and CA 125 basal level (*p* = 0.080).

### 3.3. Definition of an “Inflammatory-Score”

Based on previous findings, we generated a model by including the neutrophil count, platelet count, ALP, LDH, albumin, d-dimer, and fibrinogen, allocating 0 or 1 to lower or upper values calculated on the median, respectively. Each factor was added, and only the albumin value was subtracted based on univariate analysis. We calculated this score: (0–2) = 0; (3–4) = 1; (5–7) = 2. Firstly, we showed a better OS in patients with lower scores compared to patients with a maximum score (*p* = 0.001). This result confirmed the significant result for OS according to PFI (*p* = 0.004). We correlated this “inflammatory score” to pharmacogenomics data detected by DMET analysis. We found a strong correlation between the inflammatory score and *UGT2A1*_57885 > (rs2288741). Indeed, patients with a high inflammatory score rarely carried heterozygous *UGT2A1*_57885 > (rs2288741). Accordingly, combined heterozygous *UGT2A1*_57885 > (rs2288741) and low inflammatory score patients presented a better outcome. Next, we performed the Fisher’s exact test to evaluate the correlation between this score and platinum-sensitivity (as variable dichotomous on PFI), and the result was significant (*p* = 0.021).

At present, platinum-based regimens are the mainstay therapeutic option for EOC patients. Indeed, the lack of platinum-response remains one of the most important adverse prognostic factors for these patients. In this context, the identification of predictive biomarkers may have a crucial role in clinical practice, allowing a better patient stratification for personalized treatment. Taking into account this scenario, we afforded the identification of prognostic biomarkers starting with the common hematology evaluations routinely performed in clinical practice. Indeed, each analyte was correlated to survival outcome to define their potential prognostic role (univariate analysis). Moreover, identified factors were correlated to PFI and response to platinum treatment in order to explore the potential predictive role, while significant variables were used to perform the multivariate analysis to identify independent prognostic factors. The pharmacogenomic evaluation was included both as a literature review for the evaluation of the role of ADME genes in our issue and as a DMET analysis from a blood sample of our patients (in a random sample selection of the enrolled population). Our findings prompt some considerations. First of all, the outcome of patients treated at our institute appears to be in line with the data in the relevant literature. This evidence, despite the small sample size of our analysis, could exclude selection bias. Interestingly, our exploratory results showed that high NLR (both for median value cut-off and prognostic value reported in the literature) was correlated with a worse prognosis. Furthermore, the identification of several parameters in univariate analyses allowed us to build an inflammatory-score (including low albumin methods value, high neutrophil count, platelet count, LDH, ALP, d-dimer ad fibrinogen) which was associated with poor OS and lower platinum-response. Moreover, with the DMET^TM^ array platform, we investigated a list panel of genetic variants in 231 ADME genes, disclosing a correlation of heterozygous genotypes to OS benefit (e.g., in *ABCC3* analysis). Furthermore, we reviewed the literature in order to evaluate the coherence of our data. On this basis, it is possible to highlight that a better survival reported with several allelic variants was particularly detected in PS patients. Interestingly, ABCC3, a member of the ATP-binding-cassette (ABC) family of transporters, seems to regulate the platinum-sensitivity which might be correlated to the expression of miR-200 [38]. Conversely, *ABCB1* is one of the most important ADME genes related to a favorable outcome and it seems that it is strongly associated with ER expression and chemo-resistance [39]. *ABCB1* retains a prognostic role strongly correlated to a debulking outcome. Thus, high *ABCB1* expression correlates to better prognosis and chemo-sensitivity only in patients with the minimal residual disease [40]. Moreover, some authors demonstrated that at 3’ the untranslated region (3’-UTR) of the *ABCB1* gene contained a potential miRNA binding site for miR-186 which may regulate platinum-sensitivity by targeting *ABCB1* [41]. Instead, UGT2A1, a UDP Glucuronosyltransferase Family 2 Member A1 Complex Locus was confirmed as a potential platinum-response predictor (*p* = 0.01) with a significant prognostic role (*p* = 0.05). Interestingly, we showed that *UGT2A1* is strongly related to neutrophil count and NLR expression as a predictor of outcome. Our findings might have potential relevance for the personalized management of OC patients who underwent the platinum regimen. In conclusion, our results indicate that OC is an extremely heterogeneous disease in which several factors play a role in individual drug metabolism and drug resistance. However, the small sample size of our mono-institutional series did not allow us to build a conclusive model by including all significant variables for the design of a personalized BRLMM (Bayesian Robust Linear Model with Mahalanobis distance classifier) algorithm (which remains a future goal) for the validation set. Indeed, the major limitation of our study is the low number of patients enrolled, the retrospective design, and the lack of an independent validation series. Despite these limitations, our observation may provide a clinically supportive model given the “friendly” availability of this data. Regarding DMET analysis, we considered 30 patients as a discovery set in order to explore pharmacogenomic correlation and platinum-sensitivity with a “proof of concept” aim. We showed that the only one highly statistically significant selected SNP was rs2288741 in *UGT2A1* as a platinum-response predictor (*p* = 0.01) with a potentially significant prognostic role (*p* = 0.05). Indeed, our analysis, which warrants further investigation with a larger sample size from different institutions and different genetic backgrounds as required by the REMARK check-list [42,43], must be considered hypothesis-generating, and new studies are eagerly awaited in this field. In the era of personalized medicine, the availability of new predictive biomarkers is crucial for the selection of better treatments in the scenario of a personalized continuum of care.

## Figures and Tables

**Table 1:** **Basal characteristics of patients.** For each predefined prognostic factor, we reported a descriptive table on the number of patients and the respective percentage for a single variable.

Characteristics	Number of Patients (%)
**Median age (59 years)** - **Age between 18–59 years** - **Age > 59 years**	
	46 (51) 45 (49)
**Performance Status (PS ECOG)** - **0** - **1** - **2**	
	81 (95) 2 (2.5) 2 (2.5)
**Histological subtypes** - **high grade serous OC (HGSOC)** - **high grade endometrioid tumours.**	
	87 (95) 5 (5)
**Stage at diagnosis** - **I–II** - **III–IV**	
	11 (12) 79 (88)
**Site of disease** - **Nodes** - **Liver** - **Peritoneal inv** - **Ascites**	
	47 (53) 6 (7) 58 (64) 47 (53.4)
**Grading** - **1** - **2** - **3**	
	1 (1) 12 (13) 78 (86)
**Median value of CA125** - **≤** **377** - **>377**	
	42 (50) 42 (50)
**First line treatment (Carbo-tax)**	92 (100)
**Avastin** - **Yes** - **No**	
	23 (28) 59 (72)
**Platinum sensitivity** - **PR** - **PS**	
	25 (35) 46 (65)
**BRCA status (34)** - **BRCA wt** - **BRCA mut**	
	24 (70) 10 (30)
**BRCA status (34)** - **Yes** - **Not**	
	37 (54) 32 (46)

PS ECOG: Performance Status Eastern Cooperative Oncology Group; OC: Ovarian Cancer; HGSOC: High Grade Serous Ovarian Cancer; PR: platinum-resistant; PS: platinum-sensitive.

**Table 2:** Basal characteristics of patients, laboratory cut-off. Patients were separated into two groups on the basis of median value. Each parameter was analyzed on the median value and then reported in terms of number and percentage for each variable.

Characteristics (on Median Value)	Number of Patients (%)
**Neutrophil count** - **≤6700 cells/mm^3^** - **>6700 cells/mm^3^**	
	46 (50) 46 (50)
**Lymphocyte count** - **≤** **1275** **cells/mm3** - **>1275 cell** **s/mm^3^**	
	46 (50) 46 (50)
**Platelet count** - **≤400 × 10^3^ cells/mm^3^** - **>400 × 10^3^ cells/mm^3^**	
	46 (50) 46 (50)
**Neutrophil count** - **≤6700 cells/mm^3^** - **>6700 cells/mm^3^**	
	46 (50) 46 (50)
**NLR** - **≤6** - **>6**	
	52 (57) 40 (43)
**PLR** - **≤** **244** - **>244**	
	46 (50) 46 (50)
**Albumin** - **≤4.3 g/dL** - **>4.3 g/dL**	
	44 (48) 8 (52)
**Alkaline Phosphatase** - **≤** **81 UI/L** - **>81 UI/L**	
	48 (52) 44 (48)
**Fibrinogen** - **≤244 mg/dL** - **>244 mg/dL**	
	45 (49) 47 (51)
**D-Dimer** - **≤** **1.88 mg/L** - **>1.88 mg/L**	
	46 (50) 46 (50)
**LDH** - **≤371 UI/L** - **>371 UI/L**	
	46 (50) 46 (50)

NLR: Neutrophil to Lymphocyte Ratio; PLR: Platelet to Lymphocyte Ratio; LDH: Lactate dehydrogenase.

**Table 3:** Overall Survival (OS) for major prognostic factors. Table shows results summarised considering the impact of each prognostic factor on survival obtained by univariate analysis by Log Rank test.

Prognostic Factors	*p*-Value	OS HR (95% C.I.)	Median
**Grading**	0.06	1.81 (0.81–4.04)	52
**BRCA status**	0.18	2.68 (0.59–12.22)	72
**BRCAness**	0.00003	0.25 (0.13–0.47)	74
**Stage at diagnosis** **(I/II vs. III/IV)**	0.023	3.58 (1.1–11.6)	48
**Ascites**	0.00001	4.04 (2–7)	35
**Peritoneal** **involvement**	0.002	2.68 (1.39–5.17)	43
**Liver** **involvement**	0.036	2.64 (1.02–6.86)	37
**Node** **involvement**	0.74	1.10 (0.6–2.04)	53
**Pleural** **effusions**	0.013	2.4 (1.17–4.95)	23
**Internal** **surgery**	0.04	2.62 (1.31–5.21)	43
**Platinum** **sensitivity**	<0.00001	0.12 (0.05–0.25)	94
**Avastin** **treatment**	0.11	0.45 (0.16–1.26)	127
**Albumin**	0.02	0.47 (0.24–0.91)	72
**Neutrophil** **count**	0.22	1.48 (0.72–2.83)	43
**Lymphocyte** **count**	0.66	0.86 (0.45–1.65)	52
**Platelet** **count**	0.068	1.79 (0.94–3.40)	43
**NLR**	0.19	1.52 (0.8–2.91)	43
**PLR**	0.41	1.3 (0.68–2.48)	43
**D-Dimer**	0.05	2.01 (0.98–4.14)	43
**Fibrinogen**	0.01	2.9 (1.46–5.76)	35
**LDH**	0.02	2.15 (1.11–4.17)	30
**ALP**	0.33	1.38 (0.7–2.69)	48
**CA125**	0.14	1.59 (0.84–3.02)	42
**CA125** **decrease**	0.009	0.56 (0.29–1.10)	67

HR: Hazard Ratio; C.I.: Confidence Interval; NLR: Neutrophil to Lymphocyte Ratio; PLR: Platelet to Lymphocyte Ratio; LDH: Lactate dehydrogenase; ALP: Alkaline Phosphatase.

**Table 4:** DMET analysis (Summary of results). Genotypes of 20 SNPs in ADME genes and the association with platinum-based response and OS in patients with OC.

Chr	Gene (Genotype)	dbSNP	*p*-Value *	RR *p*-Value	PFI *p*-Value	PFS *p*-Value	OS *p*-Value
**7**	***ABCB1*** ***(CC)***	rs2235033	0.0004	0.0058	0.09	0.43	0.8
**7**	***ABCB1*** ***(AA)***	rs2235013	0.0004	0.0058	0.21	0.43	0.8
**16**	***ABCC1*** ***(TT)***	rs246221	0.001	0.013	0.025	0.065	0.028
**17**	***ABCC3*** ***(CC)***	rs2277624	0.0006	0.003	0.18	0.18	0.008
**21**	***CBR1*** ***(CC)***	rs1005695	0.003	0.046	0.84	0.36	0.074
**21**	***CBR3*** ***(AG)***	rs2835286	0.0001	0.005	0.06	0.33	0.53
**21**	***CBR3*** ***(GG)***	rs2835286	0.0002	0.01	0.057	0.26	0.19
**19**	***CYP2B6*** ***(CG)***	rs4803418	0.003	0.018	0.46	0.12	0.059
**19**	***CYP2B6*** ***(CT)***	rs4803419	0.003	0.018	0.46	0.15	0.055
**1**	***FMO4*** ***(AG)***	rs2223477	0.0005	0.04	0.21	0.38	0.7
**X**	***MAOB*** ***(AG)***	rs1799836	0.0008	0.0039	0.21	0.27	0.81
**17**	***PNMT*** ***(CC)***	rs2952151	0.003	0.018	0.10	1	0.92
**6**	***PPARD*** ***(AG)***	rs7751481	0.002	0.022	0.32	0.3	0.08
**6**	***PPARD*** ***(CT)***	rs1883322	0.002	0.046	0.18	0.3	0.03
**6**	***SLC22A2*** ***(AA)***	rs316003	0.002	0.018	0.08	0.79	0.425
**6**	***SLC22A2*** ***(GG)***	rs316003	0.003	0.005	0.21	0.78	0.6
**3**	***SLC6A6*** ***(CC)***	rs2341970	0.003	0.02	0.09	0.65	0.38
**16**	***SULT1A2*** ***(AG)***	rs11401	0.001	0.007	0.28	0.25	0.05
**4**	***UGT2A1*** ***(AC)***	rs2288741	0.0001	0.08	0.08	0.31	0.0001
**2**	***UGT1A9*** ***(TT)***	rs3821242	0.002	0.21	0.006	0.04	0.014

Chr: Chromosome; dbSNP: Single Nucleotide Polymorphism identifier based on NCBI; RR: Response Rate; PFI: Platinum-Free Interval; PFS: Progression Free Survival; OS: Overall Survival. * *p*-value corrected according to Bonferroni correction (*p*-value < 0.00313).

**Table 5:** Platinum-sensitivity multivariate analysis. Table shows the results of a multivariate analysis obtained by selecting each independent prognostic factor calculated on the basis of a univariate analysis.

Platinum-Sensitivity Factors—*p* Value	
**Albumin**	0.219
**ALP**	0.381
**CA125 (basal)**	0.058
**Neutrophil count**	0.921
**Platelet count**	0.329
**D-Dimer**	0.020
**Fibrinogen**	0.988
**LDH**	0.000
**Lymphocytes**	0.828
**NLR**	0.881
**PLR**	0.400

ALP: Alkaline Phosphatase; LDH: Lactate dehydrogenase NLR: Neutrophil to Lymphocyte Ratio; PLR: Platelet to Lymphocyte Ratio.

## Data Availability

Not applicable.
